# The lowest level of tumor involvement is a significant prognostic factor for upper tract urothelial carcinoma after radical nephroureterectomy: A large retrospective cohort study

**DOI:** 10.3389/fonc.2022.1031774

**Published:** 2022-12-01

**Authors:** Ying-Che Huang, Hung-Jen Wang, Min-Tse Sung, Yao-Chi Chuang, Yen-Ta Chen, Yuan-Tso Cheng, Chih-Hsiung Kang, Hui-Ying Liu, Yin-Lun Chang, Po-Hui Chiang, Hao-Lun Luo

**Affiliations:** ^1^ Department of Urology, Kaohsiung Chang Gung Memorial Hospital and Chang Gung University and College of Medicine, Kaohsiung, Taiwan; ^2^ Department of Pathology, Kaohsiung Chang Gung Memorial Hospital and Chang Gung University College of Medicine, Kaohsiung, Taiwan; ^3^ Center for Shockwave Medicine and Tissue Engineering, Kaohsiung Chang Gung Memorial Hospital and Chang Gung University College of Medicine, Kaohsiung, Taiwan; ^4^ College of Medicine, Kaohsiung Medical University, Kaohsiung, Taiwan; ^5^ Jhong Siao Urological Hospital, Kaohsiung, Taiwan

**Keywords:** carcinoma, transitional cell, urological neoplasms, neoplasm metastasis, urinary bladder neoplasms, nephroureterectomy

## Abstract

**Purpose:**

To evaluate the prognostic impact of the lowest level of tumor location for upper tract urothelial carcinoma (UTUC) treated with radical nephroureterectomy (RNU).

**Materials and methods:**

Data were collected from patients with UTUC treated with RNU (01/2005- 06/2020) at a single center in Taiwan. Patients were stratified by the lowest level of tumor location into three groups: renal pelvis only (RPO), above upper ureter (AUU), and below upper ureter (BUU). We compared characteristics between groups and examined the association of the lowest level of tumor involvement with intravesical recurrence (IVR), systemic metastasis (SM), and cancer-specific mortality (CSM).

**Results:**

Overall, 1239 patients (542 RPO, 260 AUU, 437 BUU) were enrolled. Concurrent bladder cancer, multifocality, tumor architecture, lymphovascular invasion, carcinoma *in situ*, and variant histology were significantly different across different tumor locations. BUU had worse five-year intravesical recurrence (IVR), systemic metastasis (SM) and cancer-specific mortality (CSM) (p < 0.001, p = 0.056 and p = 0.13, respectively). In multivariable models, the lowest level of tumor involvement was an independent predictor of IVR (AUU hazard ratio (HR) = 1.52, p = 0.007; BUU HR = 1.75, p < 0.001), but only BUU was an independent predictor of SM (HR = 1.61, p = < 0.001) and CSM (HR = 1.51, p = 0.008).

**Conclusion:**

The lowest level of tumor involvement in UTUC, especially BUU, was associated with a higher risk of IVR, SM and CSM. Assessment of the lowest level of tumor involvement after RNU may help identify patients who require more intensive follow-up.

## Introduction

Upper urinary tract urothelial carcinoma (UTUC) is a cancer of the renal pelvis and ureter lining. The incidence and biological characteristics of UTUC differ according to ethnicity and geographic region (1). The incidence of UTUC in the United States is low (2.06 cases per 100,000 person-years), accounting for only 4–5% of all UCs (2). UTUC is more common in men than in women, but female patients are more likely to have a higher cancer stage, larger tumor, and lymph node metastasis (LNM) than those of male patients (3).

The incidence and disease pattern of UTUC in Asia differs from those in Western countries. UTUC is much more common in Asian populations, and accounts for more than 10% of all UCs in Asia (4, 5). Furthermore, in Taiwan and China, UTUC is more prevalent in women than in men (4, 5), whereas female patients with UTUC have a lower proportion of high stage, large-size tumors, and LNM than those of male patients (5). Owing to the high incidence and different cancer characteristics in Asian populations, UTUC has become an important public health issue in recent years.

Radical nephroureterectomy (RNU) with bladder cuff excision is the standard treatment for patients with localized UTUC (2, 6). For patients with locally advanced UTUC, adjuvant therapies are warranted and may be added after RNU (7, 8). However, even after optimal treatment, studies have shown that a significant number of patients have disease recurrence and metastases, and die from disease progression [five-year recurrence rate, and cancer-specific mortality (CSM) of approximately 30% (9) and 30-40% (9, 10)]. Thus, identifying the predictive factors of tumor recurrence and survival is important and can be helpful for clinical assessment and decision-making.

In the 2010s, studies on patients with UTUC followed by RNU, renal pelvis tumors (RPT), and ureteral tumors (UT) did not find any significant difference in disease recurrence and mortality after adjustment for other pathologic covariates (11, 12). In those studies, however, multifocal tumors were assigned to either the renal pelvis or ureters based on the pathological stage, grade, and size of the dominant tumor, leading to biases in their conclusions. Thus, in recent years, after excluding multifocal cases, studies have observed significantly worse outcomes in patients with UT than in those with RPT (13–16). In line with recent findings on oncological outcomes, the 2020 European Association of Urology (EAU) Guidelines on UTUC also supported that tumor location was an independent prognostic factor for survival (17). However, a part of the upper ureter is surrounded by the renal parenchyma, perirenal fat, and Gerota’s fascia as the renal pelvis (18). In addition, the upper ureter and kidney have the same blood supply to the renal artery (19). These gross anatomical similarities give rise to a hypothesis that the natural course of UTUC in the upper ureter is similar to that in the renal pelvis and is quite different from that in the middle to lower ureter. Thus, this study aimed to analyze whether different ureteral tumor locations (renal pelvis, upper ureter, or middle to lower ureter) are associated with different oncological outcomes.

## Materials and methods

### Study population

This study included patients with clinically localized UTUC who underwent RNU at our institution between January 2005 and June 2020, and excluded patients who underwent nephron-sparing surgery and those with non-urothelial carcinoma histology. Finally, 1239 patients with available clinical and pathological data were included in the analysis. All patients underwent preoperative cystoscopy or computed tomography (CT) to determine the presence of concurrent bladder tumor or distant metastasis. Demographic data, such as age, sex, smoking history, concurrent bladder cancer (BCa), adjuvant therapy, disease recurrence outcome, and death, were obtained using chart review. Patients were separated into three different groups according to the lowest level of tumor location (renal pelvis tumor only, tumor above the upper ureter [defined as a ureteral tumor located above the sacroiliac joint level], and tumor below the upper ureter [defined as a ureteral tumor found at the middle to low ureter]). This retrospective study was approved by the Institutional Review Board of our hospital (IRB number: 202201112B0).

### Pathological evaluation

The diagnosis of UC was confirmed by histological analysis, and variant histology was also included in this study. Genitourinary pathologists reviewed all slides according to strictly identical criteria and were blinded to the clinical outcomes. Tumor grading was performed according to the 2004 and 2016 World Health Organization classifications (20, 21). Tumors were staged according to the Eighth American Joint Committee on Cancer (AJCC) tumor–node–metastasis (TNM) classification. Lymphovascular invasion (LVI), grade, variant histology, and concomitant carcinoma *in situ* (CIS) were assessed by a uropathologist on each representative slide.

### Follow-up protocol and definition of an oncological event

Our institutional follow-up protocol included postoperative fiber cystoscopy every three months, and renal ultrasonography every six months to assess the contralateral urinary tract. These procedures were performed during the first two years, every six months during the third year, and then annually thereafter. Abdominal CT was performed either annually or depending on the patient’s condition to assess lymph node status and local or regional recurrence of the tumor. Bone scanning, chest CT, and magnetic resonance imaging (MRI) were performed when clinically indicated. The use of postoperative bladder instillation was empirically decided by the individual urologist. Intravesical recurrence (IVR) was defined as post-nephroureterectomy urinary bladder tumor recurrence. Systemic metastasis (SM) was defined as a local recurrence or distant metastasis. Local recurrence was defined as locoregional recurrence in the ipsilateral surgical field outside the urinary tract, and distant metastasis was defined as disease recurrence outside the urinary tract and out of the locoregional surgical field. Disease in the urinary bladder or contralateral upper urinary tract was not considered indicative of SM. CSM was defined as a local recurrence or distant metastasis at the time of death.

### Statistical analysis

SPSS software (v.21) was used for statistical analyses. Chi-square or two-sample t-tests were used to analyze the distribution of between the two groups, and for intergroup comparisons. The Kaplan–Meier method with log-rank test was used to compare event-free survival between the groups. Multivariate Cox regression analysis was used to identify independent prognostic factors for oncologic outcomes. Statistical significance was set at p < 0.05, as shown in **Table 1**, and an independent association was defined as p < 0.05 in multivariate analyses, as shown in **Table 2**.

**Table 1:** Clinicopathologic Characteristics.

	Renal pelvis only	Above upper ureter	Below upper ureter	p value
	n=542	n=260	n=437
Follow up (Months)	53.7 ± 45	50.7 ± 42.4	50.8 ± 42.2	0.802
Age (years)	67.8 ± 11	67.2 ± 10.4	67.1 ± 10.3	0.456
Gender				0.266
Male	245(45.2%)	115(44.2%)	217(49.7%)	
Female	297(54.8%)	145(55.8%)	220(50.3%)	
pT stage				0.211
pT≤2	346(63.8%)	162(62.3%)	298(68.2%)	
pT>2	196(36.2%)	98(37.7%)	139(31.8%)	
Nodal status				0.377
Nx	448(82.7%)	224(86.2%)	360(82.4%)	
N0	94(17.3%)	36(13.8%)	77(17.6%)	
Laparoscopic surgery	306(56.5%)	142(54.6%)	265(60.6%)	0.236
Hydronephrosis	359(66.2%)	191(73.5%)	287(58.9%)	0.116
Smoking history	63(11.6%)	30(11.5%)	61(14%)	0.484
Concurrent BCa	38(7%)	30(11.5%)	61(14%)	<0.001
Multifocal	54(10%)	112(43.1%)	208(47.6%)	<0.001
High grade	499(92.1%)	246(94.6%)	397(90.8%)	0.199
Papillary	427(78.8%)	194(74.6%)	313(71.6%)	0.034
Lymphovascular invasion	157(29%)	82(31.5%)	86(19.7%)	<0.001
Carcinoma *in situ*	156(28.8%)	148(56.9%)	203(46.5%)	<0.001
Variant histology	192(35.4%)	85(32.7%)	107(24.5%)	<0.001
Neoadjuvant chemotherapy	9(1.7%)	3(1.2%)	7(1.6%)	0.912
Adjuvant chemotherapy	36(6.6%)	23(8.8%)	30(6.9%)	0.501
Intravesical recurrence	104(19.2%)	76(29.2%)	162(37.1%)	<0.001*/<0.001†
Metastasis	109(20.1%)	59(22.7%)	121(27.7%)	0.017*/0.056†
Cancer specific mortality	89(16.4%)	46(17.7%)	96(22%)	0.0496*/0.13†

*: Survival analyses were performed with the Cox proportional hazard model.

†: Kaplan-Meier estimate in survival analysis.

**Table 2:** Multivariate Cox regression analysis for oncological outcome.

	Intravesical recurrence				Systemic metastasis				Cancer specific mortality			
	Uni	Multi	HR	95% CI	Uni	Multi	HR	95% CI	Uni	Multi	HR	95% CI
Location												
Renal pelvis			1 (ref)				1 (ref)				1 (ref)	
Above UU	0.002	0.035	1.39	1.02-1.9	0.391	0.441	1.14	0.82-1.59	0.637	0.989	1	0.69-1.46
Below UU	<0.001	<0.001	1.59	1.22-2.09	0.017	0.005	1.5	1.13-1.99	0.048	0.049	1.37	1-1.88
Con. BCa	<0.001	<0.001	2.44	1.9-3.14	0.113				0.055			
Multifocal	<0.001	0.004	1.41	1.11-1.78	0.009	0.086	1.25	0.97-1.63	0.004	0.016	1.42	1.07-1.9
LVI	0.002	0.123	0.79	0.59-1.07	<0.001	<0.001	3.29	2.53-4.28	<0.001	<0.001	3.46	2.58-4.64
ND	0.033	0.191	0.8	0.58-1.11	0.804				0.77			
PA	0.188				<0.001	<0.001	0.57	0.44-0.73	<0.001	<0.001	0.50	0.38-0.66
CIS	0.01	0.563	1.07	0. 85-1.33	0.925				0.692			
Variant	0.005	0.047	0.78	0.6-1	<0.001	<0.001	1.66	1.3-2.11	<0.001	0.001	1.57	1.2-2.06
HG	0.294				<0.001	0.007	4.81	1.53-15.12	<0.001	0.019	5.38	1.33-21.82
Adj. CT	0.002	0.01	0.42	0.22-0.81	<0.001	0.003	1.65	1.19-2.29	<0.001	0.058	1.42	0.99-2.05
Neoadj. CT	0.815				<0.001	<0.001	3.06	1.75-5.32	<0.001	<0.001	3.11	1.7-5.69
Laparoscopic	0.249				<0.001	0.015	0.74	0.58-0.94	<0.001	0.082	0.79	0.6-1.03
Hydronephrosis	0.062				0.007	0.045	0.77	0.6-0.99	0.015	0.05	0.75	0.56-1
Smoking	0.01	0.5	1.11	0.82-1.52	0.119				0.088			
Female	<0.001	<0.001	0.64	0.5-0.8	<0.001	0.076	0.8	0.63-1.02	<0.001	0.053	0.77	0.58-1
Age>68	0.638				0.685				0.566			

UU, Upper ureter; BCa, Bladder cancer; ND, nodal dissection; LVI, Lymphovascular invasion; PA, Papillary; CIS, Carcinoma *in situ*; HG, High grade; Adj. CT, Adjuvant chemotherapy; Neoadj. CT, Neoadjuvant chemotherapy.

## Results

**Figure 1:**
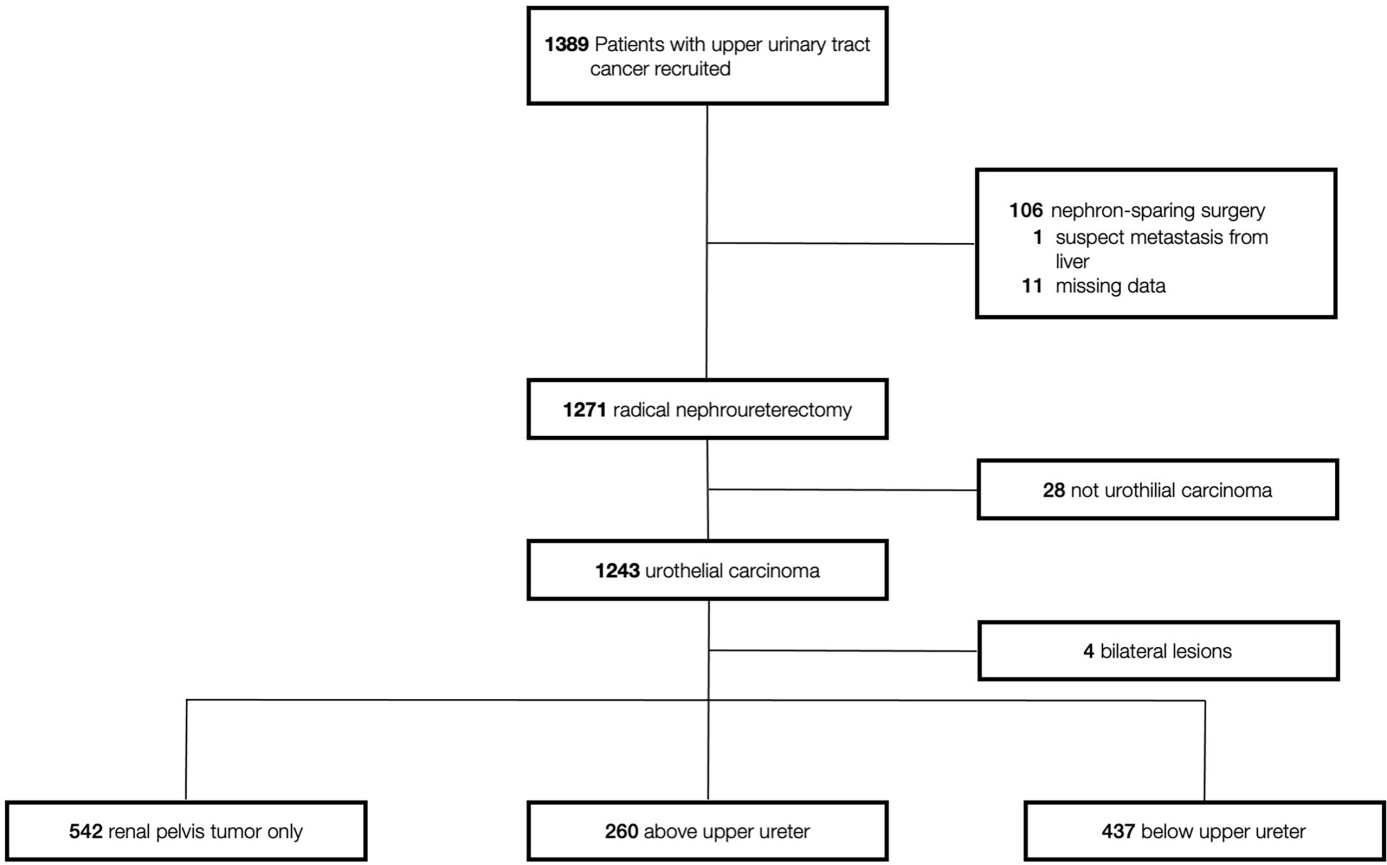
Study profile.

Of 1239 patients, tumor(s) located in the renal pelvis only (RPO), above upper ureter (AUU), and below upper ureter (BUU) were presented in 542 (43.7%), 260 (21%), and 437 (35.3%) patients, respectively (**Figure 1**). **Table 1** shows the clinical and pathological characteristics stratified by the lowest urinary tract level tumor involved. The mean follow-up durations of individuals in the RPO, AUU, and BUU groups were 53.7 ± 45, 50.7 ± 42.4, and 50.8 ± 42.2 months, respectively. The presence of concurrent BCa and multifocality was similar in AUU and BUU but lower in RPO (concurrent BCa: 11.5%, 14%, and 7%, respectively; p < 0.001; multifocality: 43.1%, 47.6%, and 10%, respectively; p < 0.001). Papillary tumor architecture was more common in RPO (78.8%) than in AUU (74.6%) or BUU (71.6%; p = 0.034). The presence of LVI and variant histology was similar in RPO and AUU but lower in BUU (LVI: 29%, 31.5%, and 19.7%, respectively; p < 0.001; variant histology: 35.4%, 32.7%, and 24.5%, respectively; p < 0.001). CIS was more common in AUU (56.9%) than in RPO (28.8%) or BUU (46.5%; p < 0.001). No significant difference was found in the rest of the clinical features.

**Figure 2:**
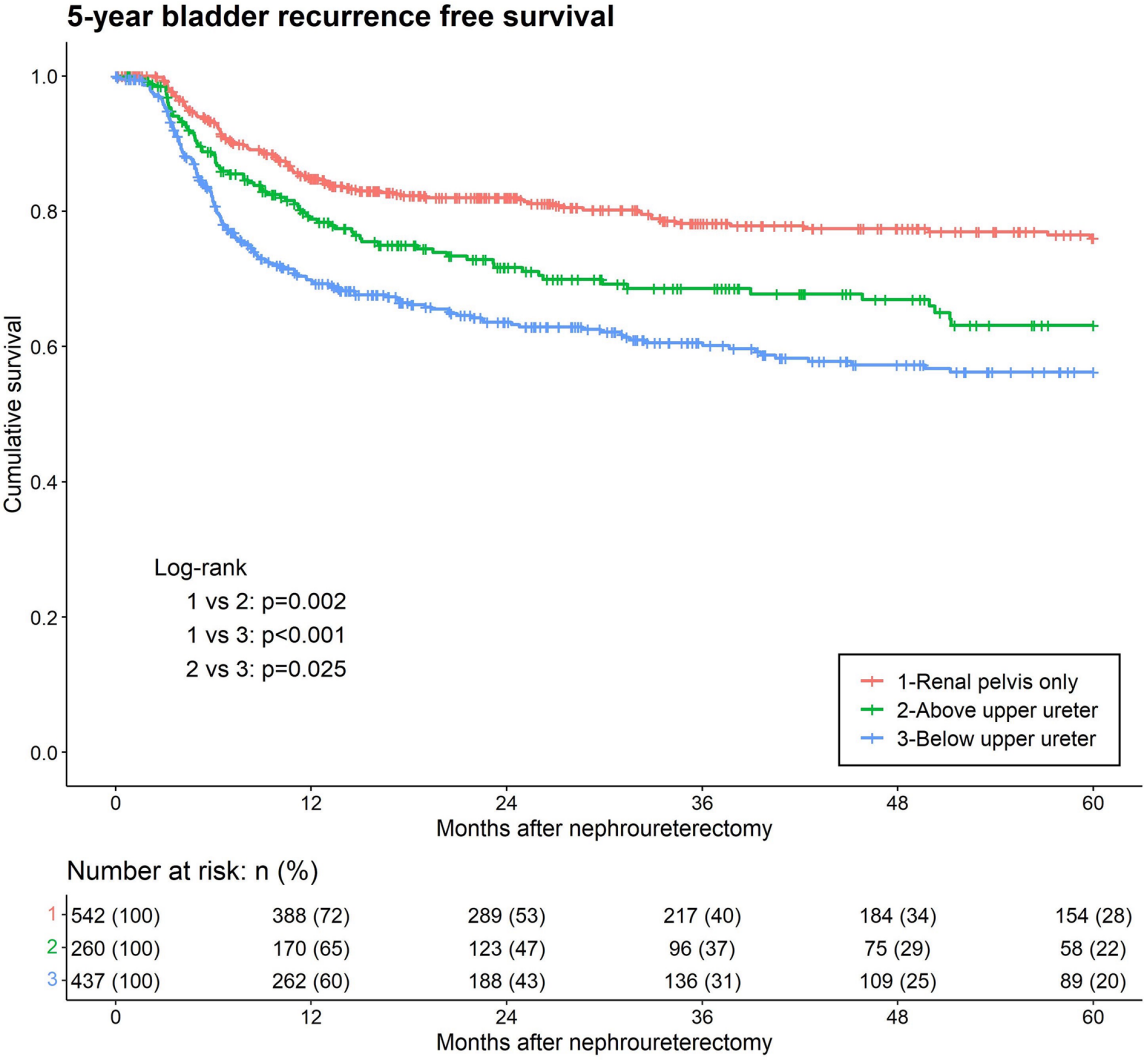
Kaplan-Meier analyses for 5-year intravesical recurrence-free survival stratified by the lowest level of tumor involvement among patients with upper tract urothelial carcinoma following rad-ical nephroureterectomy.

In our study, 342 (27.6%) patients experienced IVR. Kaplan–Meier analysis, stratified by the lowest level of tumor involvement, showed that BUU was associated with a significantly lower five-year bladder recurrence-free survival than were RPO and AUU (**Figure 2**). In the univariate analysis of IVR, the lowest level of tumor involvement, concurrent BCa, multifocality, LVI, nodal dissection (ND), CIS, variant histology, adjuvant chemotherapy, smoking, and female sex were significant prognostic risk factors for IVR. In the multivariate analysis, only the lowest level of tumor involvement (AUU hazard ratio (HR) = 1.39, p = 0.035; BUU HR = 1.59, p < 0.001), concurrent BCa, multifocality, variant histology, adjuvant chemotherapy, and female sex remained significant risk factors for IVR (**Table 2**).

**Figure 3:**
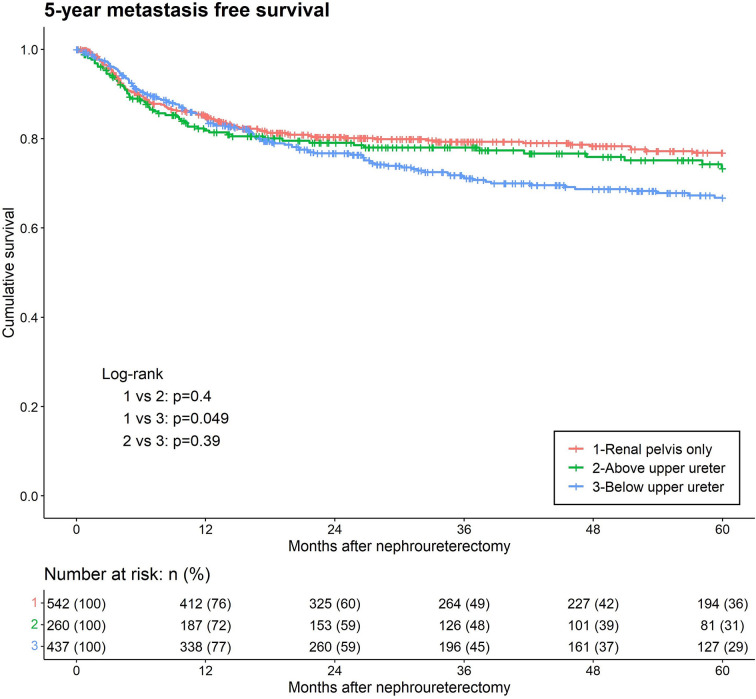
Kaplan-Meier analyses for 5-year metastasis-free survival stratified by the lowest level of tumor involvement among patients with upper tract urothelial carcinoma following radical nephroureterectomy.

During the follow-up period, distant metastases were observed in 289 (23.3%) patients. Kaplan–Meier analysis, stratified by the lowest level of tumor involvement, showed that BUU was associated with significantly lower five-year metastasis-free survival than was RPO (**Figure 3**). In the univariate analysis, BUU, multifocality, LVI, papillary (PA), variant histology, high-grade tumor, adjuvant chemotherapy, neoadjuvant chemotherapy, laparoscopic surgery, hydronephrosis, and female sex were significant prognostic risk factors for the development of SM. The results were similar in the multivariate analysis of SM, excluding multifocality and female sex, which did not contribute to SM (BUU HR = 1.5, p = 0.005) (**Table 2**).

**Figure 4:**
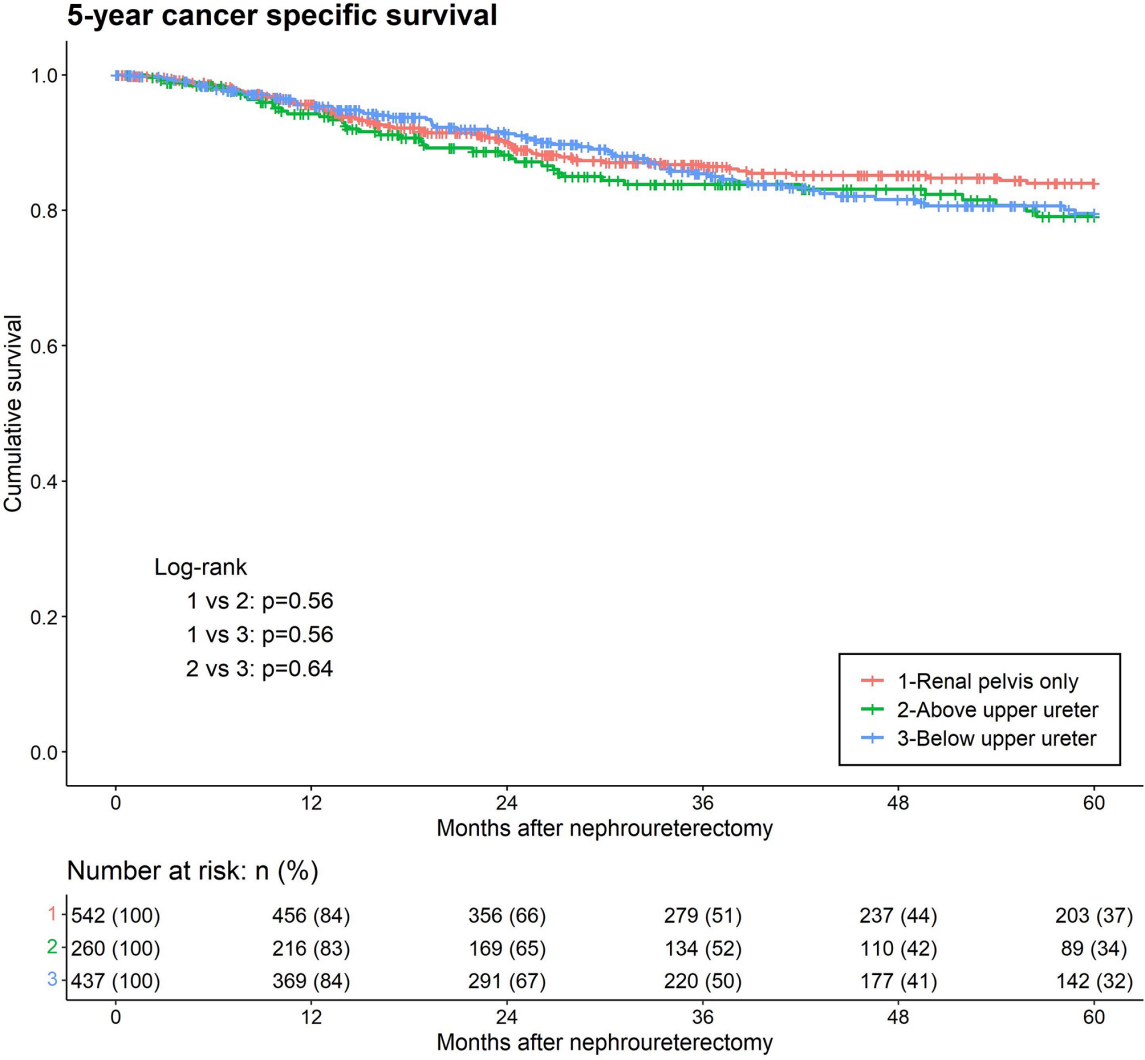
Kaplan-Meier analyses for 5-year cancer-specific survival stratified by the lowest level of tumor involvement among patients with upper tract urothelial carcinoma following radical nephroureterectomy.

Lastly, cancer-specific death from UTUC occurred in 89 (16.4%), 46 (17.7%) and 96 (22%) patients in the RPO, AUU, and BUU groups, respectively. Kaplan–Meier analysis of cancer-specific survival between the three lowest levels of tumor involvement revealed no statistical difference (**Figure 4**). In the univariate analysis for CSM, BUU, multifocality, LVI, PA, variant histology, high grade, adjuvant chemotherapy, neoadjuvant chemotherapy, laparoscopic surgery, hydronephrosis and female sex were significant prognostic risk factors for CSM. The results were similar in the multivariate analysis of CSM, excluding adjuvant chemotherapy, laparoscopic surgery and female sex, which did not contribute to CSM (BUU HR = 1.37, p = 0.049) (**Table 2**).

## Discussion

According to the latest EAU Guidelines, age, sex, smoking, a history of BCa, tumor stage, tumor grade, LVI, CIS, and variant histology are reported as significant prognostic factors for the development of UTUC. In addition to these well-established factors, tumor location has also been identified as a risk factor for poorer oncological outcomes (17). Several studies have reported that patients with UT have a significantly worse oncological outcome than patients with RPT even after adjustments for other pathological covariates (13–16). However, the surrounding structures and blood supply of the upper ureter are more similar to those of the renal pelvis than those of the middle to lower ureter. Early studies further dissecting the ureter reported that lower ureteral lesions may also result in worse prognoses. Otsuka et al. and Fang et al. found that lower ureter tumors were independent predictors of IVR after RNU in patients with UTUC (22, 23). However, the number of cases in these cohorts was too small to draw a definite conclusion. In addition, the authors of the aforementioned studies only evaluated the impact on IVR, and did not disclose other prognostic impacts, including SM and survival of lower ureteral tumors. There remains a paucity of studies on the oncological outcomes of the lowest level of tumor involvement (renal pelvis, upper ureter, or middle to lower ureter). In this study, we included demographics and various established features for adjustment to assess the impact of the lowest level of tumor involvement on IVR, SM, and CSM in UTUC. We demonstrated that tumor involvement below the upper ureter was a significant prognostic factor for worse UTUC outcomes.

In this study, we found that the lowest level of tumor involvement significantly correlated with higher IVR. Audenet et al. reported that upper tract cancer and subsequent BCa are clonally related (24). We speculate that the increased IVR rate of UTUC involving the lower ureter might be related to the distance between the tumor and the urinary bladder. Compared with the renal pelvis, the narrower space of the ureter results in a higher intraluminal pressure and urine flow rate. High pressure and fast flow can cause tumor cell detachment and seeding into the bladder (25). Furthermore, the high pressure and intraluminal tumor seeding theory also help explain the much higher top-down recurrence rate than that of bottom-up UC (15-50% vs 2-6%) (26). Otsuka et al. examined 186 patients with UTUC, where lower ureteral lesions were defined as the lowest tumor within five cm from the lower end of the ureter, and found a higher rate of IVR (HR = 1.74, p = 0.030) (22). Fang et al. evaluated 438 patients and used the same criteria as in our study, also showing that lower ureteral tumors are a predictor of bladder recurrence (HR = 1.60, p = 0.012) (23). Lastly, the surgical approach in our institution is to control the distal ureter first and prevent intraoperative tumoral detachment and subsequent seeding. Therefore, the possibility of IVR due to tumor detachment in the surgical operation is relatively low. Therefore, our finding that BUU is associated with greater IVR is supported by intraluminal seeding and intraepithelial migration theory. These theories describe that metachronous intravesical tumors clonally evolve from the nearest, transformed upper tract cancer cells (27, 28).

We observed that the SM and CSM rates of RPO and AUU were similar, whereas that of BUU was significantly higher. The difference in the SM and CSM rate between BUU and the other two groups (RPO and AUU) might result from the different anatomic structures surrounding the tumors. Waseda et al. suggested that the thin adventitia comprised of abundant blood plexus and lymphatic vessels, can advance tumor invasion (29). However, the renal pelvis and part of the upper ureter are surrounded by multiple barriers, including the renal parenchyma, perirenal fat, and Gerota’s fascia. These solid barriers tightly cover the tumor and adjacent tissues, which make the lesions completely resected by the surgeon and prevent further micro-metastasis. In contrast, the middle and distal ureters are surrounded by thin smooth muscles and weak adipose tissue. Therefore, the safe margin from normal tissue to tumor is less adequate in the BUU group than in the RPO and AUU groups (30). Taken together, the anatomical structure of BUU is associated with a soft tissue barrier that is inadequate against preventing tumor spread, and there is less of a safety margin during radical surgery, leading to a higher metastasis rate and cancer specific mortality.

A limitation of this study is that it was a retrospective cohort study in a single institution with physicians and patients related protocol deviated bias on surgical technique or density of follow-up duration. However, most patients were managed and followed according to the institutional protocol in this study. In addition, real-world patients with advanced disease may receive adjuvant chemotherapy without the same cycles because of patient preferences and tolerance of side effects. However, we included a relatively large cohort with an adequate follow-up duration, and carefully examined the prognostic impact by considering the established prognostic factors.

Our results indicate that a detailed classification of ureteral tumor location provides more prognostic information for physicians to set a more intensive follow-up and adjuvant therapy protocol. Further multi-institutional validation regarding the level of tumor involvement in patients with UTUC should be performed based on this finding, since it might improve precision clinical care.

## Conclusions

The lowest level of tumor involvement in UTUC, especially BUU, is an independent predictor of IVR, SM and CSM. Assessment of the lowest level of tumor involvement after RNU may help identify patients with a high risk of cancer recurrence who require more intensive follow-up or early administration of intravesical instillation and adjuvant therapy.

## Data availability statement

The raw data supporting the conclusions of this article will be made available by the authors, without undue reservation.

## Ethics statement

The studies involving human participants were reviewed and approved by Chang Gung Medical Foundation Institutional Review Board. Written informed consent for participation was not required for this study in accordance with the national legislation and the institutional requirements.

## Author contributions

Conception and design, Y-CH and H-LL; acquisition of data, Y-CH, H-JW, M-TS, Y-CC, Ye-TC, Yu-TC, C-HK, H-YL, Y-LC, P-HC and H-LL; analysis and interpretation of data, Y-CH and H-LL; drafting of the manuscript, Y-CH and H-LL; critical revision of the manuscript for important intellectual content, Y-CH and H-LL; statistical analysis, H-YL, Y-LC and H-LL; obtaining funding, H-LL; administrative, technical, or material support, Y-CH and H-LL; supervision, H-LL. All authors contributed to the article and approved the submitted version.

## Funding

This study was supported by grants from Chang Gung Memorial Hospital Research Project Grant CMRPG8K1091, CMRPG8K1092 and Ministry of Science and Technology, Taiwan (MOST-110-2314-B-182A-050-MY3).

## Acknowledgments

We thank Chun-Chien Hsu, Weng-Chou Yang, Wei-Ching Lee for their efforts about perioperative care and monitoring of their UTUC patients contributed in this cohort. We thank Hsin-Yi Chien, Chih-Yun Lin and the Biostatistics Center, Kaohsiung Chang Gung Memorial Hospital.

## Conflict of interest

The authors declare that the research was conducted in the absence of any commercial or financial relationships that could be construed as a potential conflict of interest.

## Publisher’s note

All claims expressed in this article are solely those of the authors and do not necessarily represent those of their affiliated organizations, or those of the publisher, the editors and the reviewers. Any product that may be evaluated in this article, or claim that may be made by its manufacturer, is not guaranteed or endorsed by the publisher.
